# *In Vitro* Selective Growth-Inhibitory Effect of 8-Hydroxyquinoline on *Clostridium perfringens* versus Bifidobacteria in a Medium Containing Chicken Ileal Digesta

**DOI:** 10.1371/journal.pone.0167638

**Published:** 2016-12-09

**Authors:** Eva Skrivanova, Filip Van Immerseel, Petra Hovorkova, Ladislav Kokoska

**Affiliations:** 1 Department of Physiology of Nutrition and Quality of Animal Products, Institute of Animal Science, Prague, Czech Republic; 2 Department of Microbiology, Nutrition and Dietetics, Faculty of Agrobiology, Food and Natural Resources, Czech University of Life Sciences, Prague, Czech Republic; 3 Department of Pathology, Bacteriology and Poultry Diseases, Ghent University, Merelbeke, Belgium; 4 Department of Crop Sciences and Agroforestry, Faculty of Tropical AgriSciences, Czech University of Life Sciences, Prague, Czech Republic; University of Catania, ITALY

## Abstract

*Clostridium perfringens*-induced necrotic enteritis is generally controlled by antibiotics. However, because of increasing antibiotic resistance, other antibacterial agents are required, preferably ones that do not affect the beneficial intestinal microbiota of the host. This study evaluated the *in vitro* selective growth-inhibitory effect of 8-hydroxyquinoline (8HQ) on *C*. *perfringens* vs. bifidobacteria in a medium containing chicken ileal digesta. Prior to the experiments, the minimum inhibitory concentrations of 8HQ and penicillin G were determined by broth microdilution assay. The minimum inhibitory concentration values of 8HQ for *C*. *perfringens* were 16–32 times lower than the values for bifidobacteria. Treatment of autoclaved and non-autoclaved chicken ileal digesta with 8HQ showed a selective anticlostridial effect. After incubation of *C*. *perfringens* with autoclaved ileal digesta for 3 h, all 8HQ concentrations tested (32–2048 μg/mL) significantly reduced *C*. *perfringens* bacterial count. In contrast, the same treatment had no or only a slight effect on bifidobacteria counts. Unlike 8HQ, penicillin G did not exhibit any selectivity. Similar results were obtained after incubation for 24 h. In non-autoclaved ileal digesta, all 8HQ concentrations tested significantly reduced *C*. *perfringens* bacterial counts after incubation for 30 min and 3 h, while no effect was observed on bifidobacteria. These results suggest that 8HQ may serve as a prospective veterinary compound for use against necrotic enteritis in poultry.

## Introduction

The control of *Clostridium perfringens*, a causative agent of avian necrotic enteritis (NE), has been of both health and economic significance to poultry production worldwide. It commonly manifests as a subclinical infection [1,2]. In addition, NetB toxin production, dietary factors, *Eimeria* co-infection, and stress are believed to be crucial factors influencing the development of NE [2]. An imbalance in the microbial community, where commensal bacteria, such as *Lactobacillus* spp. and *Bifidobacterium* spp., do not compete with pathogens for adhesion sites or nutrients, can promote intestinal colonization by pathogenic bacteria. Since the colonization and rapid proliferation of *C*. *perfringens* in the small intestine is one of the principal factors that induce NE in birds, competitive microbiota, such as bifidobacteria, can lower the risk of clostridial adhesion and thus decrease the risk of NE occurrence [3].

Until now, NE has usually been controlled using various conventional antibiotics such as bacitracin and penicillin [1,4,5]. However, their use is well known to result in the development of antibiotic resistance. This has led to the regulated use of antibiotics in both veterinary and human medicine. In addition, antibiotics are known for their ability to affect not only the target microorganism but also the beneficial microbiota in both humans and animals, including poultry [6,7].

The above-mentioned concerns have prompted efforts to develop other types of antimicrobial agents. Plant-derived compounds have been proposed as prospective alternatives to conventional antibiotics in veterinary medicine [8]. In addition, the limitations of the antibiotics commonly used for treating clostridial infections have led to a search for new agents with better anticlostridial selectivity over the normal gut microbiota [9]. One of the possible candidates is 8-hydroxyquinoline (8HQ), a quinolone alkaloid of plant origin that is currently used as a topical bacteriostatic and fungistatic agent in human medicine. The antibacterial effect of 8HQ and its derivatives has been observed *in vitro* toward *Clostridium* spp., *Escherichia coli*, and *Staphylococcus aureus* [10]. Apart from its wide range of antimicrobial properties, including antibacterial, antiviral, and antiparasitic effects [10], 8HQ has been shown to inhibit the *in vitro* growth of *Clostridium* spp., including *C*. *perfringens*; however, it exerts a significantly lower effect on bifidobacteria [11,12]. Its selective effect has been confirmed against *C*. *difficile* and *Bifidobacterium longum* subsp. *longum* in a co-culture analyzed by flow cytometry [13]. It has been found that 8HQ helps in isolating bifidobacteria from human and animal feces, especially when there is a risk of clostridial contamination [14].

Control of NE in poultry by using anticlostridial agents can be evaluated *in vivo* by using experimentally infected animal models. However, avian NE is a complex multi-factorial disease [15,16,17], in which other factors (such as co-infection with *Eimeria*, immunosuppressive viruses, or dietary modifications) contribute to its induction [17,18]. Unfortunately, those interventions may significantly affect the experimental design. Thus, to study the impact of 8HQ on *C*. *perfringens*, the effect of the anticlostridial agents can be tested *in vitro*, in a relatively controlled medium of chicken intestinal contents [19,20]. Furthermore, according to the principles of the 3 Rs (replacement, reduction, and refinement), *in vitro* techniques should be used, when possible, to reduce animal suffering and improve well-being [21].

In this context, evaluation of new selective anticlostridial agents is highly desirable for their possible use in poultry veterinary practice. Therefore, the objective of this study was to evaluate the *in vitro* selective effect of 8HQ on *C*. *perfringens* in a medium of the chicken intestinal system, and to compare its effect with that of penicillin.

## Materials and Methods

### Chemicals

With the exception of Wilkins–Chalgren anaerobic broth, soy peptone, tryptose sulfite cycloserine (TSC) agar, and egg yolk emulsion (Oxoid CZ, Brno, Czech Republic), all other chemicals (8HQ, agarose, bacitracin, buffer concentrate, cysteine, glycerol, mupirocin, penicillin G, RedTaq Ready Mix, and Tris-acetate-EDTA [TAE]) were purchased from Sigma-Aldrich (Prague, Czech Republic). The stock solution of 8HQ was prepared in dimethyl sulfoxide (DMSO, Lach-Ner, Neratovice, Czech Republic). Penicillin G was dissolved at appropriate concentrations deionized water.

### Bacterial strains and media

Three isolates of *C*. *perfringens* and 3 *Bifidobacterium* spp. strains were used in this study. *C*. *perfringens* CIP 105178 was obtained from the Collection of Institut Pasteur (Paris, France). *C*. *perfringens* CCM 4435 and *B*. *animalis* CCM 4988 were purchased from the Czech Collection of Microorganisms (Brno, Czech Republic). *C*. *perfringens* 56, a clinical isolate from a case of chicken NE, was obtained from a culture collection at Ghent University (Ghent, Belgium). This strain is resistant to rifampicin, well characterized, and shown to be suitable for inducing experimental infections [22]. *B*. *longum* TP1, a clinical isolate from human feces, was obtained from a collection of microorganisms at the Department of Microbiology, Nutrition and Dietetics at the Czech University of Life Sciences (Prague, Czech Republic). *B*. *gallinarum* DSM 20670 was obtained from the Leibniz Institute DSMZ (Braunschweig, Germany). Clostridia and bifidobacteria were both incubated and stored in Wilkins–Chalgren anaerobic broth, which was supplemented with soy peptone (5 mg/mL) and cysteine (0.5 mg/mL). For storage at −20°C, glycerol at a final concentration of 15% (v/v) was used as a cryoprotectant.

### Minimum inhibitory concentration (MIC) determination

The broth microdilution method [23], which was modified according to the recommendations proposed for effective assessment of the antimicrobial potential of natural products [24], was used for measuring 8HQ MIC in 96-well microtiter plates. Briefly, a two-fold dilution range of 8HQ in the broth was used, starting with an initial concentration of 2048 μg/mL. Each sample, excluding the negative control (no bacterial strain and no antibacterial agent in the well), was inoculated with a standardized bacterial suspension of 10^6^ CFU/mL. For inoculum standardization, the turbidity of the bacterial suspension was adjusted to 0.5 McFarland standard (1.5 × 10^8^ CFU/mL) using a Densi-La-Meter II (Lachema, Neratovice, Czech Republic) spectrophotometric device. Penicillin G (0.031–2μg/mL) was assayed in each plate as a positive control. The plates were prepared in an anaerobic chamber (Bugbox, BioTrace, Bridgend, UK) and subsequently incubated for 48 h at 37°C in an anaerobic jar (Anaerobic Plus System, Oxoid). The bacterial growth was measured in terms of the turbidity using a Multiskan Ascent microplate reader (Thermo Fisher Scientific, USA) at 405 nm. MIC was defined as the concentration that resulted in a greater than 80% inhibition of the microbial growth relative to a growth control. The tests were performed in triplicate in 3 independent experiments. The mode values were used for MIC determination. The solvents used as the negative control did not inhibit the growth of any strain tested.

### Assaying the effect of chicken ileal digesta (ID) on the growth-inhibitory activity of 8HQ

To simulate the natural intestinal conditions in chicken, the effect of 8HQ was evaluated in the presence of chicken ID, under both autoclaved (to avoid interference from background microbiota) and non-autoclaved conditions. Initially, the activity of 8HQ against each of the 6 bacterial strains was examined in the presence of autoclaved ileal contents. Subsequently, its effect on the clinical isolate *C*. *perfringens* 56 was determined in the presence of the non-autoclaved ID. Under both the autoclaved and non-autoclaved ID conditions, the inhibitory activity of 8HQ was evaluated at 4 concentrations (32, 128, 512, and 2048 μg/mL). Penicillin G was included at concentrations of 0.25, 1, 4, and 16 μg/mL as an antibiotic control in each experiment. Penicillin G was selected in accordance with a previous experiment that showed its stronger antibacterial activity compared to bacitracin. The concentration range for both 8HQ and penicillin G was selected according to the results of the previously described susceptibility test, whereas the initial concentrations correspond to the MIC values determined in the previous experiment. A positive control (an inoculated medium with ID at a 1:1 ratio, no treatment) and a negative control (a non-inoculated medium with ID at a 1:1 ratio, no treatment) were included. For the experiment using non-autoclaved contents, a growth control (an inoculated medium with no ID added) was also included within each incubation set.

### Growth-inhibitory activity of 8HQ in the presence of autoclaved chicken intestinal contents

To perform this set of experiments, the methodology of Si et al. [25] and Vasudevan et al. [26] was used, with some modifications. For each tested bacterial strain (3 clostridia and 3 bifidobacteria strains), 3 independent incubations were performed as follows: A composite sample of ileal contents, obtained from 30 clinically healthy, 35-day-old Ross 308 broiler chickens (Xavergen, Kostelec nad Cernymi lesy, Czech Republic) receiving a commercial wheat- and corn-based feed mixture free of any antimicrobial agents, was collected and aseptically placed into gas-tight glass flasks (3 g for each experimental sample) containing Wilkins–Chalgren anaerobic broth (3 mL), buffered with sodium phosphate buffer (pH 7.2). The flasks were flushed with CO_2_, closed with rubber stoppers, and autoclaved at 121°C for 15 min. After autoclaving, appropriate quantities of 8HQ or penicillin G were added. All samples, excluding the negative control, were subsequently inoculated with one of the tested bacterial strains. An inoculum was prepared for each tested strain by suspending the 24-h-grown culture in the broth to achieve a turbidity equivalent to that of the McFarland standard (10^8^ CFU/mL). Each sample was inoculated with 0.3 mL of the diluted culture (final density, 10^7^ CFU/mL). The samples were incubated anaerobically at 42°C, in triplicate. At 0, 3, and 24 h of incubation, the bacterial counts of *C*. *perfringens* and bifidobacteria were determined by plating 100 μL of the appropriate dilution (decimally diluted in sterile peptone water) on selective agar plates. For *C*. *perfringens* and *Bifidobacterium* spp. quantification, TSC agar with egg yolk and modified Wilkins–Chalgren agar with mupirocin were used, respectively [27]. Suspected bacterial colonies were confirmed by microscopy (Eclipse E200, Nikon, Nikon Instruments Europe BV, The Netherlands). Differences in bacterial counts for each treated group, compared to the positive control were determined by SAS, using t-test [28].

### Growth-inhibitory effect of 8HQ in the presence of non-autoclaved chicken intestinal contents

For this set of experiments, the modified method of Namkung et al. [29] was used. To assess the effect of 8HQ on the clinical isolate *C*. *perfringens* 56 in a medium containing the background microbiota present in fresh intestinal contents, the methodology was adjusted as follows: Thirty composite samples of ileal contents were diluted at a 1:1 ratio, as described above. After adding the appropriate quantities of 8HQ or penicillin G, all samples, excluding the negative control, were inoculated with rifampicin-resistant *C*. *perfringens* 56 at the same final density as in the previous experiment. In this assay, the rifampicin-resistant *C*. *perfringens* bacterial count was determined, in addition to the total *C*. *perfringens* and bifidobacteria counts (representing the background microbiota). The concentrations of 8HQ and penicillin G were similar to those in the previous experiment; however, the incubation periods were changed to 30 min and 3 h. Rifampicin-resistant *C*. *perfringens* growth was evaluated on Wilkins–Chalgren agar containing 20 mg/mL of rifampicin. The total *C*. *perfringens* count was determined on Wilkins–Chalgren agar with no rifampicin added. The *Bifidobacterium* spp. count was determined after incubation for 24 h on modified Wilkins–Chalgren agar with mupirocin [27]. The results were analyzed statistically as described above. The bacterial colonies were confirmed by microscopy. In the case of *C*. *perfringens*, suspected colonies were further confirmed by PCR on an iQ5 thermocycler (BioRad, USA), using RedTaq Ready Mix primers. The PCR conditions were adjusted according to the methodology of Wise and Siragusa [30]. The template used for the PCR procedure was prepared as described by Kanakaraj et al. [31] with a few modifications: Each suspected colony was suspended in 50 μL of sterile distilled water, boiled for 10 min, and centrifuged at 10,000 *g* for 5 min. The supernatant was used as a template for the PCR reaction. The amplified products were visualized by electrophoresis in 2% agarose in TAE buffer at 100 V for 45 min.

## Results

In this study, the *in vitro* selective growth-inhibitory effect of 8HQ on clostridia was determined by broth microdilution and 2 other assays based on incubation in the presence of autoclaved and non-autoclaved ileal contents.

The broth microdilution test results (Table 1) revealed a clear selective growth-inhibitory effect of 8HQ on the 3 tested strains of *C*. *perfringens* (MIC = 32 μg/mL), compared to the 3 bifidobacteria strains (MICs = 512 and 1,024 μg/mL). Penicillin G exerted a stronger, but nonselective, clostridial/bifidobacterial growth-inhibitory effect, where the growth of all tested strains was inhibited most frequently at a concentration of 0.25 μg/mL, with the exception of *B*. *longum* TP1 (MIC = 0.125 μg/mL).

**Table 1:** *In Vitro* Antibacterial Effect of 8-Hydroxyquinoline (8HQ), Penicillin G toward *C*. *perfringens* and Selected Bifidobacteria.

Bacterium	MIC^a^^,^ ^b^ (μg/mL)	
	8HQ	Penicillin G
***C*. *perfringens* 56**	32	0.25
***C*. *perfringens* CCM 4435**	32	0.25
***C*. *perfringens* CIP 105178**	32	0.25
***B*. *longum* TP1**	1024	0.125
***B*. *animalis* CCM 4988**	512	0.25
***B*. *gallinarum* DSM 20670**	512	0.25

^a^Minimum inhibitory concentration, i.e. the median value of tested concentrations, resulting in a greater than 80% inhibition of growth relative to a growth control.

^b^Results of 3 independent experiments, each performed in triplicate.

The effect of 8HQ and penicillin G on *C*. *perfringens* (3 strains), *B*. *longum*, *B*. *animalis*, and *B*. *gallinarum* was evaluated further in a medium containing autoclaved chicken ileal contents (Tables 2 and 3). After an incubation period of 3 h (Table 2), all tested concentrations of 8HQ (32–2048 μg/mL) significantly reduced the count of *C*. *perfringens* (*p* ≤ 0.01) compared to the untreated control (8HQ = 0 μg/mL). In contrast, the same treatment had no significant effect on the *B*. *longum* count. In the case of *B*. *gallinarum* and *B*. *animalis*, only the highest concentration of 8HQ (2048 μg/mL) showed significant bacterial reduction (*p* ≤ 0.01). Conversely, penicillin G did not act selectively, and led to a significant reduction in the growth of all tested bacteria (*p* ≤ 0.01). After incubation for 24 h, the antibacterial activity was similar to the activity observed after incubation for 3 h, with the exception of *B*. *animalis*, where 8HQ significantly reduced the bacterial growth (*p* ≤ 0.01) at the 2 highest concentrations after 24 h (Table 3). In addition, treatment of *C*. *perfringens* CIP 105178 with the highest concentration of 8HQ reduced the bacterial counts below the detection limit (2 log_10_ CFU/g).

**Table 2:** Effects of 8-Hydroxyquinoline (8HQ) and Penicillin G on Autoclaved Ileal Contents of Broiler Chickens Artificially Inoculated with *C*. *perfringens* and Selected Bifidobacteria (CFU/g)^a^ after Incubation for 3 h at 42°C.

Treatment	Concentration (μg/mL)	*C*. *perfringens* 56	*C*. *perfringens* CCM 4435	*C*. *perfringens* CIP 105178	*B*. *longum* TP1	*B*. *animalis* CCM 4988	*B*. *gallinarum* DSM 20670
**8HQ**	32	6.27 ± 0.25*	6.35 ± 0.35*	6.62 ± 0.59*	7.46 ± 0.33	7.07 ± 0.50	6.95 ± 0.16
	128	5.60 ± 0.36*	5.75 ± 0.26*	6.48 ± 0.50*	7.65 ± 0.11	6.84 ± 0.78	6.95 ± 0.07
	512	4.75 ± 0.36*	4.8 ± 0.34*	4.89 ± 0.59*	7.28 ± 0.44	6.85 ± 0.83	6.75 ± 0.28
	2048	3.63 ± 0.06*	3.71 ± 0.34*	3.70 ± 0.72*	6.97 ± 0.86	6.60 ± 0.21*	6.17 ± 0.21*
**Penicillin G**	0.25	5.85 ± 0.59*	5.90 ± 0.17*	5.98 ± 0.29*	7.30 ± 0.29	6.95 ± 0.33	6.69 ± 0.86*
	1	5.58 ± 0.80*	5.52 ± 0.54*	4.73 ± 0.38*	6.94 ± 0.34*	6.67 ± 0.35*	6.42 ± 0.75*
	4	5.75 ± 0.74*	5.83 ± 0.41*	4.57 ± 0.51*	6.65 ± 0.37*	6.34 ± 0.35*	6.73 ± 0.95*
	16	4.74 ± 0.81*	5.56 ± 0.54*	4.05 ± 0.10*	6.56 ± 0.49*	6.40 ± 0.15*	6.17 ± 0.32*
**Control groups**							
**Positive control**^**b**^	0	7.37 ± 0.54	7.39 ± 0.34	7.39 ± 0.53	7.67 ± 0.32	7.08 ± 0.34	7.16 ± 0.38
**Negative control**^**c**^	0	< 2*	< 2*	< 2*	< 2*	< 2*	< 2*

^a^Colony-forming units, means ± SD of 3 independent experiments.

^b^Inoculated samples with no 8HQ or penicillin G added to the incubation mixture.

^c^Non-inoculated samples with no 8HQ or penicillin G added to the incubation mixture.

^*^Values in the same column differ significantly from the positive control (*p* ≤ 0.01).

**Table 3:** Effects of 8-Hydroxyquinoline (8HQ) and Penicillin G on Autoclaved Ileal Contents of Broiler Chickens Artificially Inoculated with *C*. *perfringens* and Selected Bifidobacteria (CFU/g)^a^ after Incubation for 24 h at 42°C.

Treatment	Concentration(μg/mL)	*C*. *perfringens* 56	*C*. *perfringens* CCM 4435	*C*. *perfringens* CIP 105178	*B*. *longum* TP1	*B*. *animalis* CCM 4988	*B*. *gallinarum* DSM 20670
**8HQ**	32	7.46 ± 0.48^*^	7.09 ± 0.35^*^	5.24 ± 0.28^*^	9.26 ± 0.30	9.81 ± 0.09	9.02 ± 0.08
	128	6.48 ± 0.60^*^	5.70 ± 0.43^*^	4.30 ± 0.61^*^	9.27 ± 0.44	9.83 ± 0.12	9.09 ± 0.12
	512	4.34 ± 0.37^*^	5.59 ± 0.36^*^	3.39 ± 0.53^*^	9.16 ± 0.43	8.35 ± 0.11^*^	8.79 ± 0.36
	2048	3.43 ± 0.51^*^	3.78 ± 0.3^*^	< 2^*^	8.50 ± 0.34	7.31 ± 0.36^*^	6.02 ± 0.74^*^
**Penicillin G**	0.25	3.43 ± 0.56^*^	3.53 ± 0.92^*^	2.78 ± 0.77^*^	4.11 ± 0.19^*^	5.71 ± 0.27^*^	5.62 ± 0.43^*^
	1	3.16 ± 0.15^*^	2.93 ± 0.83^*^	< 2^*^	4.19 ± 0.37^*^	5.22 ± 0.42^*^	5.51 ± 0.19^*^
	4	2.72 ± 0.12^*^	2.43 ± 0.38^*^	< 2^*^	3.74 ± 0.28^*^	5.05 ± 0.35^*^	5.73 ± 0.35^*^
	16	2.43 ± 0.51^*^	< 2^*^	< 2^*^	3.79 ± 0.74^*^	4.16 ± 1.04^*^	5.86 ± 0.11^*^
**Control groups**							
**Positive control**^**b**^	0	8.90 ± 0.35	9.07 ± 0.32	9.09 ± 0.62	9.04 ±0.07	9.75 ± 0.26	9.03 ± 0.06
**Negative control**^**c**^	0	< 2^*^	< 2^*^	< 2^*^	< 2^*^	< 2^*^	< 2^*^

^a^Colony-forming units, means ± SD of 3 independent experiments.

^b^Inoculated samples with no 8-HQ or penicillin G added to the incubation mixture.

^c^Non-inoculated samples with no 8-HQ or penicillin G added to the incubation mixture.

^*^Values in the same column differ significantly from the positive control (*p* ≤ 0.01).

All tested concentrations of 8HQ and penicillin G significantly reduced (*p* ≤ 0.01) the counts of both total and rifampicin-resistant *C*. *perfringens* when tested in a medium containing non-autoclaved ID (Tables 4 and 5). With the exception of the lowest concentration (0.25 μg/mL), the bifidobacteria count was significantly reduced after incubation for 30 min in the presence of penicillin G (*p* ≤ 0.01). In contrast, 8HQ did not affect the bifidobacterial count at any of the concentrations tested (Table 4). Furthermore, 2 concentrations of 8HQ (512 and 2048 μg/mL) reduced *C*. *perfringens* counts below the detection limit. Incubation for 3 h (Table 5) did not change the pattern of 8HQ action. All 8HQ concentrations tested significantly reduced clostridial growth (*p* ≤ 0.01) while showing no significant effect on bifidobacteria. In contrast to the selective effect of 8HQ, penicillin G at the highest concentration tested (16 μg/mL) significantly reduced both clostridia and bifidobacteria counts (*p* ≤ 0.01).

**Table 4:** Effects of 8-Hydroxyquinoline (8HQ) and Penicillin G on Non-Autoclaved Ileal Contents of Broiler Chickens Artificially Inoculated with *C*. *perfringens* 56 (CFU/g)^a^ after Incubation for 30 min at 42°C.

Treatment	Concentration (μg/mL)	*C*. *perfringens* total	*C*. *perfringens* (rifampicin-resistant)	*Bifidobacterium* spp.
**8HQ**	32	6.82 ± 0.57^*^	6.52 ± 0.73^*^	6.94 ± 0.71
	128	3.55 ± 0.51^*^	3.43 ± 0.71^*^	6.86 ± 0.58
	512	< 2^*^	< 2^*^	6.96 ± 0.77
	2048	< 2^*^	< 2^*^	6.56 ± 0.79
**Penicillin G**	0.25	6.14 ± 0.31^*^	6.23 ± 0.41^*^	6.20 ± 0.72
	1	6.02 ± 0.23^*^	6.15 ± 0.42^*^	5.88 ± 0.34^*^
	4	6.04 ± 0.49^*^	6.03 ± 0.86^*^	5.96 ± 0.56^*^
	16	5.79 ± 0.45^*^	5.82 ± 0.50^*^	5.86 ± 0.35^*^
**Control groups**
**Positive control**^**b**^	0	7.67 ± 0.56	7.55 ± 0.44	6.47 ± 0.92
**Negative control**^**c**^	0	3.27 ± 0.37^*^	< 2^*^	6.59 ± 0.59
**Growth control**^**d**^	0	8.12 ± 0.59	8.17 ± 0.68	< 2^*^

^a^Colony-forming units, means ± SD of 3 independent experiments.

^b^Medium with digesta (1:1), inoculated with *C*. *perfringens* 56.

^c^Medium with digesta (1:1), no bacterial inoculation.

^d^Medium with no digesta added, inoculated with *C*. *perfringens* 56.

^*^Values in the same column differ significantly from the positive control (*p* ≤ 0.01).

**Table 5:** Effects of 8-Hydroxyquinoline (8HQ) and Penicillin G on Non-Autoclaved Ileal Contents of Broiler Chickens Artificially Inoculated with *C*. *perfringens* 56 (CFU/g)^a^ after Incubation for 3 h at 42°C.

Treatment	Concentration (μg/mL)	*C*. *perfringens* total	*C*. *perfringens* (rifampicin-resistant)	*Bifidobacterium* spp.
**8HQ**	32	2.77 ± 1.17*	2.69 ± 1.10*	5.91 ± 0.87
	128	2.29 ± 0.68*	2.28 ± 0.63*	5.98 ± 0.78
	512	< 2*	< 2*	5.66 ± 0.91
	2048	< 2*	< 2*	5.94 ± 0.86
**Penicillin G**	0.25	5.40 ± 0.48*	5.44 ± 0.47*	5.93 ± 0.63
	1	5.83 ± 0.16*	5.63 ± 0.47*	6.52 ± 0.43
	4	5.14 ± 0.25*	4.90 ± 0.49*	6.26 ± 0.75
	16	3.27 ± 0.43*	< 2*	5.38 ± 0.57*
**Control groups**
**Positive control**^**b**^	0	8.13 ± 0.26	8.12 ± 0.35	6.28 ± 0.85
**Negative control**^**c**^	0	3.7 ± 0.82*	< 2*	6.59 ± 0.79
**Inoculum**^**d**^	0	8.84 ± 0.75	8.77 ± 0.69	< 2*

^a^Colony-forming units, means ± SD of 3 independent experiments.

^b^Medium with digesta (1:1), inoculated with *C*. *perfringens* 56.

^c^Medium with digesta (1:1), no bacterial inoculation.

^d^Medium with no digesta added, inoculated with *C*. *perfringens* 56.

*Values in the same column differ significantly from the positive control (*p* ≤ 0.01).

## Discussion

The present study focused on evaluating the *in vitro* selective growth-inhibitory effect of 8HQ on *C*. *perfringens* and bifidobacteria in the complex environment of chicken ID. Prior to testing, 8HQ MIC values for *C*. *perfringens* and bifidobacteria were determined using the broth microdilution assay. In accordance with previous reports [11–14, 32], our results showed a notable selective inhibitory effect of 8HQ on *C*. *perfringens* compared to its effect on bifidobacteria. The susceptibility of all strains tested was similar to that reported by Novakova *et al*. [12], with a slight difference in the case of *B*. *longum*. The newly tested *B*. *gallinarum* exhibited similar resistance to 8HQ as other bifidobacteria strains. Our results are supported by those of Kim *et al*. [11] and Jeon *et al*. [32]; however, these authors used different methods for determining the anticlostridial/bifidobacterial effect of 8HQ.

Apart from the experimental infection of model animals, *in vitro* cultures of intestinal contents can serve as a method for evaluating bacterial susceptibility to agents used in animal health management [21]. In previous reports, the anticlostridial activity of several classes of natural compounds, such as butyrate glycerides, caprylic acid, and essential oils, was measured in media containing ID [25,26,29]. In the present study, 8HQ exerted growth-inhibitory effects on various strains of *C*. *perfringens* in media containing autoclaved and non-autoclaved ileal contents. To our knowledge, this is the first study to assess the effect of 8HQ on a representative sample of beneficial microflora and clostridia simultaneously using this methodology.

The mechanism of 8HQ selectivity has not been clarified. Chelating activity is exhibited by 8HQ has a chelating activity, where it scavenges the metallic cations from its environment while reactive oxygen species are formed. Metal ions play an important role in biological processes, and metal homeostasis is required for maintenance of cellular functions [10]. Chelated metals become unavailable, inhibiting certain metabolic processes [33]. For instance, RNA synthesis is inhibited if bivalent cations required for RNA polymerase activity are chelated [34]. In relation to our previous findings [13], it could be suggested that 8HQ causes selective RNA polymerase inhibition because of the different capacities of bifidobacteria and clostridia to accumulate metallic ions. It is likely that the oxidative stress defense systems of these bacterial species also vary. The role of metalloenzymes in these species should explored, to identify the mechanism of 8HQ selective antibacterial action [13]. In our study, the inhibitory effect of 8HQ was particularly notable in non-autoclaved ileal contents, with marked effects after 30 min and 3 h of incubation. One possible reason could be the presence of a thermolabile compound with antibacterial activity (such as a bacterocin, or other antimicrobial protein-based structure) [35]. However, this assumption should be investigated prior to forming any conclusions.

In regards to 8HQ safety, the Toxicology Data Network (TOXNET, US National Library of Medicine) reports a moderate toxicity based on oral exposure in rats (LD_50_ = 1.2 g/kg). There is currently no evidence of carcinogenity. Except in high doses, 8HQ is not known to exert toxicity in humans. Moreover, a recent study of 8HQ found low toxicity in murine peritoneal macrophages and no hemolytic activity in human red blood cells [36]. A preparation containing 8HQ was patented in 1989 as an oral antimicrobial agent (administered in feed or water) for use in poultry [37]. In veterinary practice, a commercial 8HQ sulfate-containing ointment is used for post-milking teat disinfection in dairy cattle [38]. Some halogenated 8HQ derivatives, used for treating amoebiasis in humans, are considered comparatively benign, except for frequent allergic reactions [39]. However, clioquinol, originally used as an antimicrobial agent for amoebiasis (traveler´s diarrhea), was withdrawn from oral use in some countries after reports of its neurotoxicity [10, 40].

A study of the pharmacokinetics of 8HQ administered intravenously in rats found biotransformation to glucuronide and sulfate metabolites, followed by excretion in the urine and bile [41]. The fate of orally administered 8HQ is considered similar, based on the findings of studies on clioquinol [42]. However, more detailed studies of 8HQ should be performed in poultry, including a thorough toxicity determination. Furthermore, the effect of 8HQ on the total intestinal microbiome should be clarified in future experiments.

## Conclusion

In summary, 8HQ showed *in vitro* selectivity against *C*. *perfringens* over bifidobacteria when evaluated using a broth microdilution method and assays based on its incubation in media containing chicken ID. Our results, supported by literature-based toxicological data, suggest that 8HQ can serve as a prospective anticlostridial agent for the treatment and prevention of NE in poultry. However, studies that focus on its *in vivo* anticlostridial activity and safety are necessary before implementing its application in veterinary practice. Molecular biology techniques should be used to gain insights into the effect of 8HQ on the complex intestinal.

## References

[1] Gadbois P, Brennan JJ, Bruce HL, Wilson JB, Aramini JJ. The role of penicillin G potassium in managing *Clostridium perfringens* in broiler chickens. Avian Dis. 2008; 52: 407–411. 10.1637/8114-091807-Reg 18939627

[2] Timbermont L, Haesebrouck F, Ducatelle R, Van Immerseel F. Necrotic enteritis in broilers: an updated review on the pathogenesis. Avian Pathol. 2011; 40: 341–347. 10.1080/03079457.2011.590967 21812711

[3] Fuller R. The chicken gut microflora and probiotic supplements. J Poult Sci. 2001; 38: 189–196.

[4] Butaye P, Devriese LA, Haesebrouck F. Antimicrobial growth promoters used in animal feed: effects of less well-known antibiotics on Gram-positive bacteria. Clin Microbiol Rev. 2003; 16: 175–188. 10.1128/CMR.16.2.175-188.2003 12692092 PMC153145

[5] Agunos A, Léger D, Carson C. Review on antimicrobial therapy of selected bacterial diseases in broiler chickens in Canada. Can Vet J. 2012; 53: 1289–1300. 23729827 PMC3500121

[6] Clemente JC, Ursell LK, Parfrey LW, Knight R. The impact of the gut microbiota on human health: an integrative view. Cell. 2012; 148: 1258–1270. 10.1016/j.cell.2012.01.035 22424233 PMC5050011

[7] Ubeda C, Pamer EG. Antibiotics, microbiota, and immune defense. Trends Immunol. 2012; 33: 459–466. 10.1016/j.it.2012.05.003 22677185 PMC3427468

[8] Cheng GY, Hao HH, Xie SY, Wang X, Dai MH, Huang LL, et al Antibiotic alternatives: the substitution of antibiotics in animal husbandry? Front Microbiol. 2014; 5: 69–83. 24860564 10.3389/fmicb.2014.00217 PMC4026712

[9] Chen C, Dolla NK, Casadei G, Bremner JB, Lewis K, Kelso MJ. Diarylacylhydrazones: Clostridium-selective antibacterials with activity against stationary-phase cells. Bioorg Med Chemi Lett. 2014; 24: 595–600. 10.1016/j.bmcl.2013.12.015 PMC3912389 24360560

[10] Prachayasittikul V, Prachayasittikul S, Ruchirawat S, Prachayasittikul S. 8-Hydroxyquinolines: a review on their metal cheating properties and medicinal applications. Drug Des Devel Ther. 2013; 7: 1157–1178. 10.2147/DDDT.S49763 24115839 PMC3793592

[11] Kim YM, Jeong EY, Lim JH, Lee HS. Antimicrobial effects of 8-quinolinol. Food Sci Biotechnol. 2006; 15 (5): 817–819.

[12] Novakova J, Vlkova E, Bonusova B, Rada V, Kokoska L. *In vitro* selective inhibitory effect of 8-hydroxyquinoline against bifidobacteria and clostridia. Anaerobe. 2013; 22: 134–136. 10.1016/j.anaerobe.2013.05.008 23770542

[13] Novakova J, Dzunkova M, Musilova S, Vlkova E, Kokoska L, Moya A, et al Selective growth-inhibitory effect of 8-hydroxyquinoline towards *Clostridium difficile* and *Bifidobacterium longum* subsp. *longum* in co-culture analyzed by flow cytometry combined with fluorescent in situ hybridization. J Med Microbiol. 2014; 63: 1663–1669. 10.1099/jmm.0.080796-0 25298160

[14] Novakova J, Vlkova E, Salmonova H, Pechar R, Rada V, Kokoska L. Anticlostridial agent 8‐hydroxyquinoline improves the isolation of faecal bifidobacteria on modified Wilkins–Chalgren agar with mupirocin. Lett Appl Microbiol. 2016; 62: 330–335. 10.1111/lam.12552 26849418

[15] Collier CT, Hofacre CL, Payne AM, Anderson DB, Kaiser P, Mackie RI, et al Coccidia-induced mucogenesis promotes the onset of necrotic enteritis by supporting *Clostridium perfringens* growth. Vet Immunol Immunopathol. 2008; 122: 104–115. 10.1016/j.vetimm.2007.10.014 18068809

[16] Park SS, Lillehoj HS, Allen PC, Park DW, Fitz Coy S, Bautista DA, et al Immunopathology and cytokine responses in broiler chickens coinfected with *Eimeria maxima* and *Clostridium perfringens* with the use of an animal model of necrotic enteritis. Avian Dis. 2008; 52: 14–22. 10.1637/7997-041707-Reg 18459290

[17] Lee KW, Lillehoj HS, Jeong W, Jeoung HY, An DJ. Avian necrotic enteritis: Experimental models, host immunity, pathogenesis, risk factors, and vaccine development. Poult Sci. 2011; 90: 1381–1390. 10.3382/ps.2010-01319 21673152

[18] Shojadoost B, Vince AR, Prescott JF. The successful experimental induction of necrotic enteritis in chickens by *Clostridium perfringens*: a critical review. Vet Res. 2012; 43: 1–12. 23101966 10.1186/1297-9716-43-74 PMC3546943

[19] Si W, Gong J, Han Y, Yu H, Brennan J, Zhou H, et al Quantification of cell proliferation and α-toxin gene expression of *Clostridium perfringens* in the development of necrotic enteritis in broiler chickens. Appl Environ Microbiol. 2007; 73: 7110–7113. 10.1128/AEM.01108-07 17827329 PMC2074978

[20] Pang Y, Patterson JA, Applegate TJ. The influence of copper concentration and source of ileal microbiota. Poult Sci. 2009; 88: 586–592. 10.3382/ps.2008-00243 19211529

[21] Baumans V. Use of animals in experimental research: an ethical dilemma? Gene Ther. 2004; 11: 64–66. 10.1038/sj.gt.3302371 15454959

[22] Timbermont L, Lanckriet A, Dewulf J, Nollet N, Schwarzer K, Haesebrouck F, et al Control of *Clostridium perfringens*-induced necrotic enteritis in broilers by target-released butyric acid, fatty acids and essential oils. Avian Pathol. 2010; 39: 117–121. 10.1080/03079451003610586 20390546

[23] Hecht DW. Susceptibility testing of anaerobic bacteria In: Murray P.R., E.J., Baron MA, Pfaller FC, Tenover RH, Yolken (Eds.), Manual of Clinical Microbiology, 7th ed. ASM Press, Washington, D.C., USA; 1999 pp. 1555–1562.

[24] Cos P, Vlietinck AJ, Berghe DV, Maes L. Anti-infective potential of natural products: How to develop a stronger in vitro ‘proof-of-concept’. J Ethnopharmacol. 2006; 106: 290–302. 10.1016/j.jep.2006.04.003 16698208

[25] Si W, Ni X, Gong J, Yu H, Tsao R, Han Y, et al Antimicrobial activity of essential oils and structurally related synthetic food additives towards *Clostridium perfringens*. J Appl Microbiol. 2009; 106: 213–220. 10.1111/j.1365-2672.2008.03994.x 19054237

[26] Vasudevan P, Marek P, Nair MKM, Annamalai T, Darre M, Khan M, Venkitanarayanan K. *In vitro* inactivation of *Salmonella* Enteritidis in autoclaved chicken cecal contents by caprylic acid. J Appl Poult Res. 2005; 14 (1): 122–125.

[27] Rada V, Petr J. A new selective medium for the isolation of glucose nonfermenting bifidobacteria from hen caeca. J Microbiol Meth. 2000; 43: 127–32. 10.1016/s0167-7012(00)00205-0 11121611

[28] SAS Institute. SAS System for Windows, Release 8.2. SAS Institute, Cary, NC, 2001.

[29] Namkung H, Yu H, Gong J, Leeson S. Antimicrobial activity of butyrate glycerides toward *Salmonella* Typhimurium and *Clostridium perfringens*. Poult Sci. 2011; 90: 2217–2222. 10.3382/ps.2011-01498 21934003

[30] Wise MG, Siragusa GR. Quantitative detection of *Clostridium perfringens* in the broiler fowl gastrointestinal tract by real-time PCR. Appl Environ Microbiol. 2005; 71: 3911–3916. 10.1128/AEM.71.7.3911-3916.2005 16000804 PMC1169009

[31] Kanakaraj R, Harris DL, Songer JG, Bosworth B. A multiplex PCR assay for detection of *Clostridium perfringens* in faeces and intestinal contents of pigs and in swine feed. Vet Microbiol. 1998; 63: 29–38. 9810619 10.1016/s0378-1135(98)00229-6

[32] Jeon JH, Lee CH, Lee HS. Antimicrobial activities of 2-methyl-8-hydroxyquinoline and its derivatives against human intestinal bacteria. J Korean Soc Appl Biol Chem. 2009; 52: 202–205.

[33] Chobot V, Drage S, Hadacek F. Redox properties of 8-quinolinol and implications for its mode of action. Nat Prod Commun. 2011; 6(5): 597–602. 21615015

[34] Fraser RSS, Creanor J. The mechanism of inhibition of ribonucleic acid synthesis by 8-hydroxyquinoline and the antibiotic lomofungin. Biochem J. 1975; 147: 401–10. 810137 10.1042/bj1470401 PMC1165465

[35] Jung W.J, Mabood F, Souleimanov A, Zhou X., Jaoua S, Kamoun F, et al Stability and antibacterial activity of bacteriocins produced by *Bacillus thurigiensis* and *Bacillus thurigiensis* spp. *kurstaki*. J Microbiol Biotechnol. 2008 18 (11): 1836–1840. 19047829 10.4014/jmb.0800.120

[36] Duarte MC, dos Reis Lage LM, Lage DP, Mesquita JP, Salles BCS, Lavorato SN, et al An effective *in vitro* and *in vivo* antileishmanial activity and mechanism of action of 8-hydroxyquinoline against *Leishmania* species causing visceral and tegumentary leishmaniasis. Vet Parasitol. 2016; 217: 81–88. 10.1016/j.vetpar.2016.01.002 26827866

[37] Magyar K, Varga J, Ferenc S, nee Lauko HS, Fekete P, Romvary A, et al., Synergistic veterinary composition and/or fodder premix and process for preparing same. U.S. Patent No. 4,871,722. Washington, DC: U.S. Patent and Trademark Office. 1989.

[38] Fox LK, Norell RJ. *Staphylococcus aureus* colonization of teat skin as affected by postmilking teat treatment when exposed to cold and windy conditions. J Dairy Sci. 1994; 77: 2281–2288. 10.3168/jds.S0022-0302(94)77171-X 7962850

[39] Gosselin RE, Smith RP, Hodge HC. Clinical Toxicology of Commercial Products. 5th ed. Baltimore: Williams and Wilkins; 1984 p. II–383.

[40] Tateishi J. Subacute myelo-optico-neuropathy: clioquinol intoxication in humans and animals. Neuropathology. 2000; 20: Suppl S20–S24.41. Kiwada H, Hayashi M, Fuwa T, Awazu S, Hanano M. The pharmacokinetic study on the fate of 8-hydroxyquinoline in rat. Cell. Pharm. Bull. 1977; 25 (7): 1566–1573. 409509 10.1248/cpb.25.1566

[41] Sawada Y, Hayashi M, Awazu S, Hanano M. In vivo and in vitro fates of 8-hydroxyquinoline derivatives in rat. Chem. Pharm. Bull. 26 (5): 1357–1363. 96945 10.1248/cpb.26.1357

[42] Yoshinari K, Sakamoto M, Senggunprai L, Yamazoe Y. Clioquinol is sulfated by human jejunum cytosol and SULT1A3, a human-specific dopamine sulfotransferase. Toxicology Lett. 2011; 206 (2): 229–233. 10.1016/j.toxlet.2011.07.023 21820498

